# Quantifying the Variability of Forest Ecosystem Vulnerability in the Largest Water Tower Region Globally

**DOI:** 10.3390/ijerph18147529

**Published:** 2021-07-15

**Authors:** Siqi Sun, Yihe Lü, Da Lü, Cong Wang

**Affiliations:** 1 State Key Laboratory of Urban and Regional Ecology, Research Center for Eco-Environmental Sciences, Chinese Academy of Sciences, P.O. Box 2871, Beijing 100085, China; m15510761127@163.com (S.S.); dalv_st@rcees.ac.cn (D.L.); congwang@rcees.ac.cn (C.W.); 2 University of Chinese Academy of Sciences, Beijing 100049, China

**Keywords:** forest vulnerability, Qinghai-Tibet Plateau, SPCA, forest vulnerability index

## Abstract

Forests are critical ecosystems for environmental regulation and ecological security maintenance, especially at high altitudes that exhibit sensitivity to climate change and human activities. The Qinghai-Tibet Plateau—the world’s largest water tower region—has been breeding many large rivers in Asia where forests play important roles in water regulation and water quality improvement. However, the vulnerability of these forest ecosystems at the regional scale is still largely unknown. Therefore, the aim of this research is to quantitatively assess the temporal–spatial variability of forest vulnerability on the Qinghai-Tibet Plateau to illustrate the capacity of forests to withstand disturbances. Geographic information system (GIS) and the spatial principal component analysis (SPCA) were used to develop a forest vulnerable index (FVI) to assess the vulnerability of forest ecosystems. This research incorporates 15 factors covering the natural context, environmental disturbances, and socioeconomic impact. Results indicate that the measure of vulnerability was unevenly distributed spatially across the study area, and the whole trend has intensified since 2000. The three factors that contribute the most to the vulnerability of natural contexts, environmental disturbances, and human impacts are slope aspect, landslides, and the distance to the farmland, respectively. The vulnerability is higher in forest areas with lower altitudes, steeper slopes, and southerly directions. These evaluation results can be helpful for forest management in high altitude water tower regions in the forms of forest conservation or restoration planning and implementation towards sustainable development goals.

## 1. Introduction

Forest ecosystems, which cover about one-third of the global land area, are among the most biologically rich and genetically diverse ecosystems on the Earth [1]. The common forest ecosystem services to be listed are timber, non-timber forest products, wildlife habitat, water quantity and quality, carbon sequestration and storage, recreational opportunities, life-support, and the climatizing function of a cooling and later warming effect [2]. Among them, forests play a highly important role in carbon storage for about 76–78% of the organic carbon in global terrestrial ecosystems [3]. Forests are of great significance for mitigating carbon emissions and regulating regional and global climate stability. However, researchers have argued that the forests are vulnerable to the changing climate, occurrence of extreme climatic events, and human-induced disturbances [4,5].

Mountain ranges and plateaus, the major freshwater sources for sustaining the downstream environmental and human water demands, are called the world’s “water towers” [6,7]. Forests in these water tower regions function critically for provisioning the water-related ecosystem services besides the above-mentioned carbon-related services. Unfortunately, the most important water towers are also found to be among the most vulnerable, subject to significant disturbances from climatic and socioeconomic changes [7]. However, the vulnerability of the forest ecosystems in these water tower regions remains largely unknown.

The Qinghai-Tibet Plateau (QTP), located in southwestern China, is the world’s largest water tower region and feeds many large rivers in Asia and benefits billions of people [7]. The forests of QTP that cover 12.5% of the region are experiencing potential ecological risks that may exacerbate their vulnerability. First, QTP is the largest ecologically fragile area in China with the characteristics of high altitudes, low temperatures and precipitation, simple ecosystem structure, and weak resistance and susceptibility to environmental changes [8]. Second, forest ecosystems are mainly distributed in the southeastern part of the QTP, where they are more severely disturbed by human activities such as the expansion of infrastructure [9] and wood extraction [10]. Researchers have shown that human disturbances in some areas will even reduce forests’ net primary productivity [8,11,12]. Moreover, the natural conditions of the southeastern QTP, comprising mainly high mountains and deep valleys, are conducive to the development of natural disasters such as landslides. The number and scale of these disasters are huge, and they often form hazard chains, which have extremely serious impacts on the natural ecosystems and social economy of the region [13]. Disasters such as landslides and mudflows occur frequently in forest areas [14]. Therefore, the forest ecosystems tend to be vulnerable under the influence of the natural and social disturbances, which will have a profound impact on the future distribution, productivity, and health of the forests.

Assessing the forests’ vulnerability has become a prerequisite and an important basis for smart forest management decision-making. In recent years, various methods and models have been applied for the assessment of forests’ vulnerability. The vegetation parameter of net primary production (NPP) was often used to describe the changes in ecosystem sensitivity and vulnerability [15]. However, this method cannot fully consider background factors such as climate and human interference. Some scholars calculated the vulnerability of forest ecosystems by constructing an index system [16], and many other methods used to integrate these factors have been proposed, such as the comprehensive evaluation method, the fuzzy evaluation method [17,18], the gray system evaluation method [19], the artificial neural-network evaluation method [20], the osculation value method, and the landscape metrics based evaluation method [21]. However, the variables required for these methods are sometimes difficult to acquire and apply, especially for large remote areas such as the QTP. For example, the artificial neural network evaluation model requires a large amount of historical data, and some evaluation models cannot quantify the contribution rates of each influencing factor.

Therefore, to integrate the multi-source influencing factors of vulnerability and the multi-dimensional evaluation method, we choose spatial principal component analysis (SPCA) as the main integration method for forest vulnerability assessment. SPCA is an organic combination of the principal component analysis (PCA) method and GIS, in which GIS can map the spatial attributes and distribution characteristics of multiple variables, but it cannot quantify the relationship between them [21,22,23]. PCA, or factor analysis, is a robust statistical analysis technique that reduces the dimensionality of data and extracts the innate relationship by developing composite variables [24]. Thus, the combined method of SPCA can quantitatively address the spatiotemporal variability of forest vulnerability.

Our study built an FVI using remote sensing, GIS, and SPCA to evaluate the forest ecosystem’s vulnerability in the QTP. Our objectives were as follows:

(1) Detect where and during which time periods forests are likely becoming increasingly vulnerable to natural context stress, environmental disturbances, and socioeconomic impacts.
(2) Map forest vulnerability across the QTP region and delimit the degree of protection required in different areas based on the FVI.
(3) Understand the behavior of the vulnerability relative to its driving factors.

An FVI at spatial and temporal scales relevant to land management must be regularly updated to provide managers with knowledge about where and when forests are under stress so that proactive remedial actions can be prioritized better to maintain, or even improve, forests’ health and resilience.

## 2. Materials and Methods

### 2.1. Study Site

**Figure 1:**
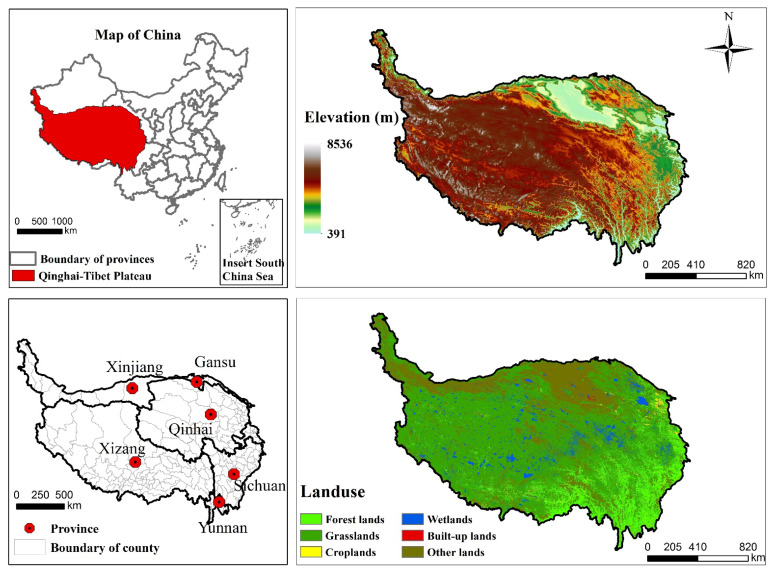
Location of the Qinghai-Tibet Plateau in China.

The Qinghai-Tibet Plateau, located in western part of China (25°59′–39°49′ N, 73°29′–104°40′ E; Figure 1), extends over 2.54 × 10^6^ km^2^ and occupies almost 26% of the total area of China. The mean altitude approximately 4430 m. Permafrost extends 40% of the QTP (above around 6000 m above sea level). Due to the special alpine environment, this region is highly sensitive to climate change, and the growing temperature rate is higher than the global average [25]. A series of changes have taken place in vegetation characteristics to adapt to the high elevation, cold and high radiation. The vegetation types from west to east are alpine grasslands, meadow grasslands, shrubs, subtropical coniferous forests, subtropical evergreen broad-leaved forests, and mid-temperate deciduous broad-leaved forests. The interior of the Qinghai-Tibet Plateau is almost dominated by grassland, while forests are mainly distributed in the southeast and marginal areas. Among them, the average altitude of alpine grassland is the highest (about 4906 m), and its average annual temperature is about 4.6 °C. The average altitude of forest areas is about 3470 m, and their annual average temperature is about 8.2 °C. Due to the special climate environment, the population density of the different provinces that make up the Qinghai-Tibet Plateau differ greatly. For example, Xizang has about 3 persons per km^2^, however, Sichuan has a population density of nearly 200 persons per km^2^.

The total forest land, covering approximately 12.5% of the region, is dominated by Pinaceae (*Pinus densata* et al.), Cupressaceae (*Sabina convallium* et al.), Fagaceac (*Lithocarpus cleistocarpus* et al.), and Betulaceae (*Betula platyphylla* et al.), etc. The forest ecosystem spans a large vertical gradient with large undulations, ranging from 391–4900 m, which are surrounded by the Hengduan Mountains, Tanggula Mountains, Qilian Mountains, and Himalayas, etc. Compared with the central part of the QTP, there is a higher average annual temperature (7.4 °C/year) and annual precipitation (612 mm/year), which belongs to the southeastern Tibet temperate humid plateau monsoon climate.

According to different geographical locations, various topographical conditions, and daily fluctuation in temperature, such climate conditions can weather rocks extremely fast. As a result, this region is prone to soil erosion, landslides, and mountain hazards. At the same time, the complex habitat conditions allow the forest ecosystem to nurture a variety of human welfare. Therefore, protecting the forest of the QTP is not only for the biodiversity habitat, but also for the healthy and sustainable development of human society.

### 2.2. Data Collection and Processing

**Figure 2:**
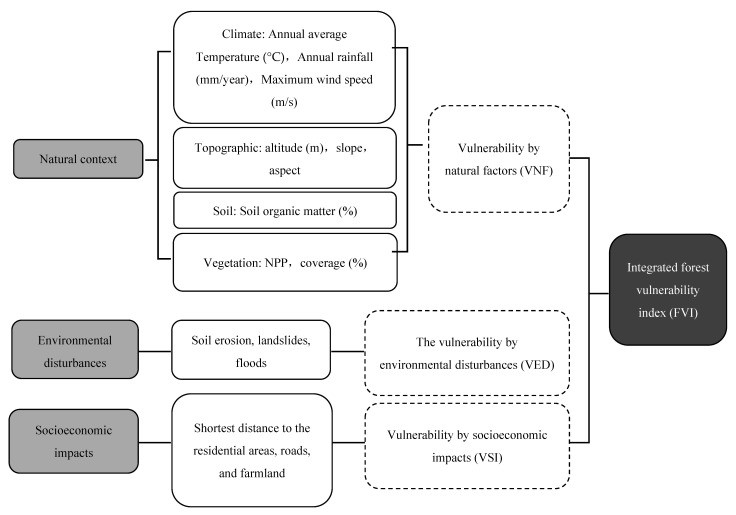
Schematic representation of the forest vulnerability evaluation.

The data analyzed in this paper (Figure 2) were compiled from a digital elevation model (spatial resolution of 90 m × 90 m provided by the Geospatial Data Cloud site, Computer Network Information Center, Chinese Academy of Sciences, http://www.gscloud.cn (accessed on 8 August 2020)); climate statistics are from meteorological stations (http://www.nmic.cn/ (accessed on 16 August 2020)); data on land use and land cover (provided by the Institute of Geographic Sciences and Natural Resources Research, CAS). Other data included the boundary of QTP, NPP data, forest coverage data, road map data, soil organic matter data, and points of geological disaster data were provided by the Institute of Geographic Sciences and Natural Resources Research, CAS (http://www.resdc.cn/ (accessed on 8 August 2020)).

The monitoring data from meteorological stations were used to produce the raster data of annual average temperature, annual precipitation, and annual maximum wind speed by using the Kriging interpolation method in ArcGIS 10.3. Slope and aspect raster data were generated from DEM by the slope and aspect tools of ArcGIS 10.3. The shortest distances to the residential areas, roads, and farmland were calculated by the Euclidean distance method. Finally, we unified all the raster data resolutions to 250 m × 250 m. The calculation of soil erosion was an assessment by an empirical erosion model named Revised Universal Soil Loss Equation (RUSLE). The model can be mathematically expressed as [26]:

(1) A=R×K×LS×C×P 

where A is the mean annual soil loss rate (tha^−1^year^−1^), R is the rainfall erosivity (MJmm.m^−2^h^−1^), K is the soil erodibility factor (dimensionless), LS is slope length and slope steepness factor (dimensionless), C is the cover and management factor (dimensionless), and P is the support practice factor (default is 1 in this article).

The method of landslide assessment revolves around the determination of some important landslide risk factors (i.e., hazard possibility, slope, rainfall, DEM, geomorphic type, NDVI, distance to the road, and river net). According to the topographic and climatic characteristics of the forest ecosystem on the QTP, as well as the weight assignment of different factors in the previous literature [27], the impact of different factors on landslides was integrated and a landslide risk map was formed by ArcGIS 10.3.

The risk of floods is mapped by a GIS-based spatial multi-index model. One part is the construction of multi-index system, another one is an analysis procedure in ArcGIS. We assigned weights to different influencing factors based on the characteristics of the study area and the existing literature on flood risk assessment. Eventually, the index system was included by vegetation coverage, river network density, population density, GDP, slope, DEM, and precipitation.

In order to remove the influence of unit differences among different indices, the normalization of selected indicators was implemented following the methodology developed for the Human Development Index (HDI) in 1990, which has also been widely used in the standardization of indicators in forest vulnerability assessment in recent years [3,16,28]. The following mathematical equation is used when an indicator has a positive functional relationship with vulnerability:

(2) $$Y_{ij}=\frac{X_{ij}-X_{min,j}}{X_{max,j}-X_{min,j}}\;\;$$

However, if an indicator has a negative functional relationship with vulnerability, then normalization of the indicator is done by following equation:

(3) $$Y_{ij}=\frac{X_{max,j-}X_{ij}}{X_{max,j}-X_{min,j}}\;\;\;\;\;\;\;\;$$

where $Y_{ij}$ is the standardized value of index *j* in grid cell *i* and varies from 0 to 1, $X_{ij}$ is the measured value of variable *j* in grid cell *i*, and $X_{max,j}$ and $X_{min,j}$ are the maximum and minimum values of index *j* in grid cell *i*, respectively.

### 2.3. Framework of Index System for Forest Vulnerability Evaluation

Based on some previous qualitative analyses of environmental features in the study area, we selected 15 independent factors representing the environmental variability to assess the forests’ vulnerability for the study area. Figure 2 shows an integrated evaluation criteria system. Natural contexts, including climate, topography, soil, and vegetation, are important determinants of vulnerability evaluation. Precipitation and temperature are the key factors for the quality of the environment, and affect the soil and forest ecosystem services, which represent the environmental situation [29]. The difference of elevation can impact vegetation evapotranspiration, regional climate, soil types, soil erosion and also affect forest vulnerability [23]. Therefore, we selected slope, aspect, and elevation to determine the vulnerability arising from topographic factors. Soil organic matter was a fundamental soil factor and key element for the growth of vegetation [30]. NPP and vegetation coverage have reflected the spatial heterogeneity of forest biomass and were important parameters that affect forest vulnerability [31].

Soil erosion, landslides, and floods are considered environmental disturbances because the study area is severely suffering from these earth surface processes and environmental problems. The occurrence of natural disasters had direct and powerful damage to the forest ecosystem. Therefore, the judgment of natural disaster risk was an important basis for forest vulnerability assessment [21].

The regional forests’ vulnerability is also strongly related to socioeconomic impact, since human activities can greatly influence the evolution of numerous environmental characteristics, and the socioeconomic impact is formed by the distance to residential areas, roads, and farmlands. These variables can represent human–environment interactions (such as economic development and environmental pollution), which can reflect the degree of human disturbance on the forest ecosystem.

### 2.4. Operation of Indices and Factors

As a general guideline, a positive correlation between the value awarded and vulnerability was employed. Then, rating maps of the vulnerability by natural factors (VNF), environmental disturbances (VED), and socioeconomic impacts (VSI) were generated for 2000, 2010, and 2015 for SPCA-based evaluation. The relevant coefficient matrix of each variable was calculated, and the eigenvalue of the matrix and its corresponding eigenvectors were computed. The results of SPCA eigenvalues are shown in Table 1. This was followed by linear grouping of eigenvectors and the computation of principal components [22,24]. The final vulnerability for three factors was graded as:

(4) Fi=R1Y1+R2Y2+R3Y3+⋯+RnYn

where Fi is i th factor of forest vulnerability, Rn represents the contribution ratio of principal component Yn, and n is the significant number of the remaining principal components. The higher the pixel value, the stronger the vulnerability.

(5) $$Ri=\frac{\lambda_{i}}{\sum_{i=1}^{n}\lambda_{i}}$$

where Ri is the contribution ratio of the i th principal component, and $\lambda_{i}$ is the eigenvalue of the i th principal component.

For the accurate evaluation of three factors vulnerability, the contribution of each of the variables was calculated [22]:

(6) $$C=\frac{\sum_{j=1}^{n}\mu_{I}^{J}\lambda_{J}}{\sum_{I=1}^{n}\sum_{j=1}^{n}\mu_{I}^{J}\lambda_{J}}\;$$

where $\lambda_{J}$ is eigenvalue of the J th principal component, and $\mu_{I}^{J}$ is the I th corresponding eigenvector of J th principal component.

Combining the vulnerability assessment results of three factors, the forest vulnerability index (FVI) was evaluated based on the distance index model. The FVI reflects a multi-dimensional space, and the minimum vulnerability of a factor is treated as a spatially determinate point used to calculate the distances between each of the other points and the minimum point; consequently, the forest vulnerability result is determined based on these distances [23]. The shorter the distance, the better the forest will be and vice versa.

(7) $$\mathrm{FVI}=\sqrt{{\left(VNF-VNF_{min}\right)}^{2}+{\left(VED-VED_{min}\right)}^{2}+{\left(VSI-VSI_{min}\right)}^{2}}\;\;\;$$

where VNF, VED, and VSI represent the vulnerability of nature context factors, environmental disturbances factors, and socioeconomic factors, respectively, and $VNF_{min}$, $VED_{min}$, and $VSI_{min}$ represent the minimum vulnerabilities of nature context factors, environmental disturbances factors, and socioeconomic factors, respectively.

VNF, VED, VSI, and FVI values were divided into five categories based on the natural break classification method [22,32,33,34], namely, *slightly vulnerable*, *moderately vulnerable*, *vulnerable*, *highly vulnerable*, *and extremely vulnerable*.

### 2.5. Method for Determining Ecological Protection-Oriented and Vulnerability-Based Spatial Pattern of Forest Protection

**Figure 3:**
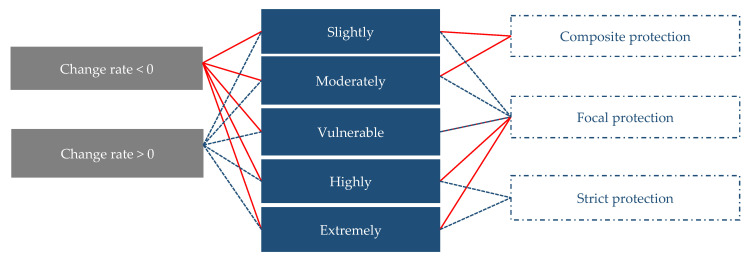
A framework for spatial conservation optimization for forest vulnerability.

The forest vulnerability assessment was designed to provide decision makers with recommendations to improve environmental quality. Therefore, we combined the change rate of forest vulnerability from 2000 to 2015 and the spatial distribution of forest vulnerability in 2015 to formulate optimal spatial protection zoning (Figure 3). The map of the optimal spatial division was represented as three regions: (1) strict protection region, (2) focal protection region, and (3) composite protection region.

## 3. Results

### 3.1. Spatiotemporal Characteristics of Forest Vulnerability Relevant to Natural and Human Factors

**Figure 4:**
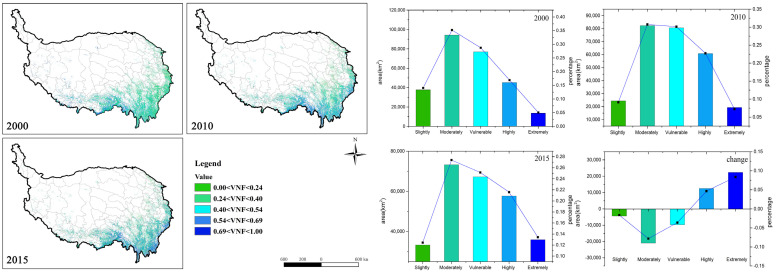
VNF spatial distribution (**left**) and area statistics of the five vulnerability levels (**right**) from 2000 to 2015. The histogram represents the area of each level, and the line chart represents the percentage area of each level.

The forest vulnerability by natural factors (VNF) intensified to a certain extent from 2000 to 2015, and the mean VNF value changed from 0.41 to 0.46 (Table 5). The forests in southern and western Tibet were more vulnerable than those in Sichuan and Yunnan provinces, which were dominated by *moderately vulnerable* and *vulnerable* levels. However, 13% of *slightly vulnerable*, *moderately vulnerable*, and *vulnerable* forest areas were transferred to *highly* and *extremely vulnerable* in the past 15 years, resulting in an increase in overall vulnerability (Figure 4). Among them, the *moderately vulnerable* had the largest reduction (7.8%), followed by the *vulnerable* (3.6%). The *highly vulnerable* and the *extremely vulnerable* levels have increased by 4.7% and 8.3%, respectively. Although part of the local forest vulnerability has increased, the *moderately vulnerable* proportion of the total area was still the largest. However, there was still a potential aggravated risk of vulnerability. From the effect of different variables, the highest contributing factor to the VNF was ASPECT (52.66%), followed by PERCIPITAION (18.33%) and TEMPERATURE (13.27%) (Table 2). Thus, it is clear that the aspect, annual mean precipitation, and temperature were the dominant factors.

**Figure 5:**
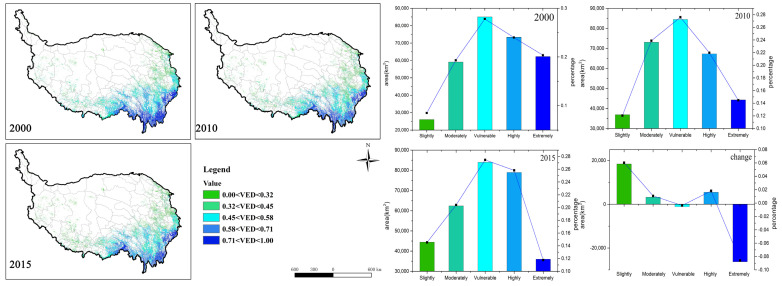
VED spatial distribution (**left**) and area statistics of the five vulnerability levels (**right**) from 2000 to 2015. The histogram represents the area of each level, and the line chart represents the percentage area of each level.

The spatial and temporal variations of environmental disturbances vulnerability (VED) are shown in Figure 5, and the mean VED value did not change significantly (0.56 to 0.52) (Table 5). During the study period, VED showed a higher forest vulnerability on the boundary of the eastern and southern parts of the QTP than the inside ones, especially in the Yunnan and Sichuan regions. However, the forest vulnerability of the southern edge significantly decreased during 2010–2015. The statistical data show that the proportion of the *slightly vulnerable* increased during 2000–2015, while the *extremely vulnerable* proportion decreased, and other vulnerability levels of forest areas did not change significantly, which suggests that the environmental disturbances’ vulnerability has been slightly alleviated. Landslides showed the greatest contribution rates to VED (Table 3). Their main influencing factors included geological conditions, precipitation, rivers, roads, and vegetation. Compared with the interior of the QTP, the boundary part showed a richer precipitation, and the topography fluctuates greatly, which makes it prone to natural disasters.

**Figure 6:**
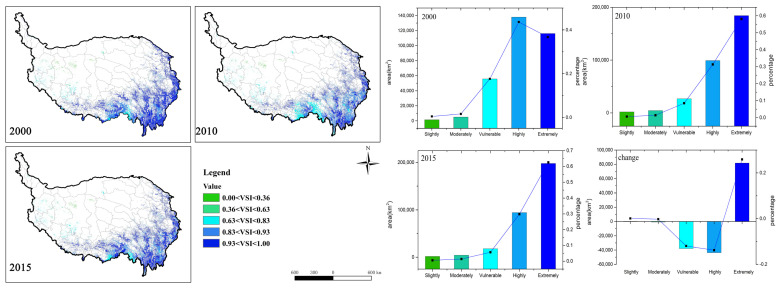
VSI spatial distribution (**left**) and area statistics of the five vulnerability levels (**right**) from 2000 to 2015. The histogram represents the area of each level, and the line chart represents the percentage area of each level.

Figure 6 illustrates the results of the vulnerabilities of socioeconomic factors (VSI). With the change of the VSI mean value from 0.89 to 0.92 (Table 5), the overall vulnerability has deteriorated during the recent 15 years. The spatial distribution shows that the *highly* and the *extremely* vulnerable categories occupy almost 80–90% of the whole forest area. Serious vulnerability areas were mainly distributed in places close to buildings, farmlands, and roads. The temporal series indicates that changes of the *moderately*, *highly*, and *extremely vulnerable* ones were in opposite trends. The *highly* and the *moderately vulnerable* proportions significantly decreased (25.8%), while the *extremely vulnerable* proportion greatly increased (25.8%). Changes on the *slightly vulnerable* and the *moderately vulnerable* were not obvious. According to the C values (Table 4), farmland (45.09%) and building construction (43.56%) both have higher influences on forest vulnerability, which are distributed through intersections with forest patches.

### 3.2. Comprehensive Forest Vulnerability in the QTP

**Figure 7:**
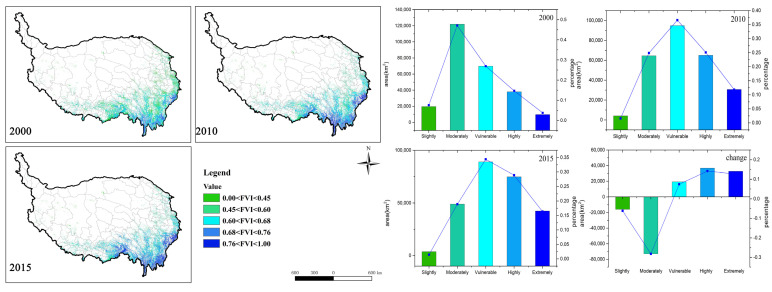
FVI spatial distribution (**left**) and area statistics of the five vulnerability levels (**right**) from 2000 to 2015. The histogram represents the area of each level, and the line chart represents the percentage area of each level.

The FVI results were graded as shown in Figure 7, with larger values representing higher vulnerability. The FVI was classified into five levels of vulnerability: *slightly vulnerable* (<0.45), *moderately vulnerable* (0.45–0.60), *vulnerable* (0.60–0.68), *highly vulnerable* (0.68–0.76), and *extremely vulnerable* (>0.76). It can be recognized from the spatial distribution that the worsening conditions of forests located in southeastern Tibet, northern Yunnan, and western Sichuan were exacerbated from 2000 to 2015.

**Figure 8:**
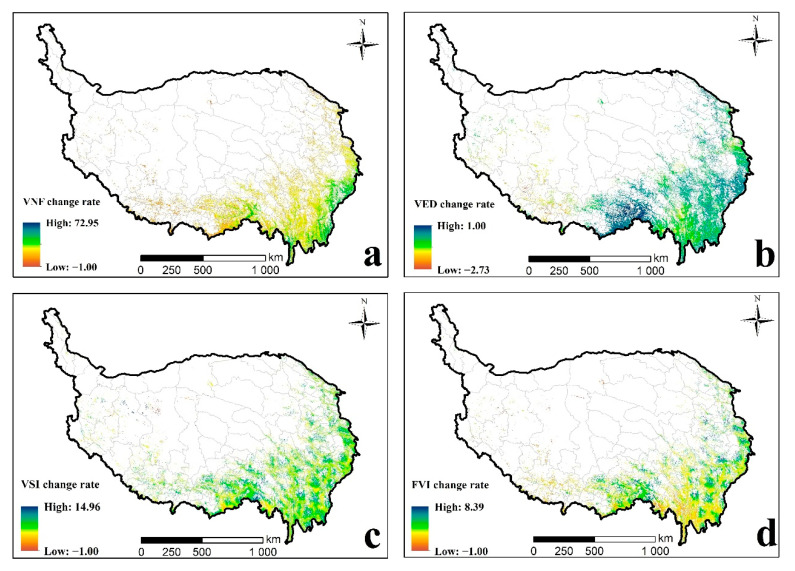
Spatial distribution of VNF change rate (**a**), VED change rate (**b**), VSI change rate (**c**), and FVI change rate (**d**) from 2000 to 2015.

From more accurate statistical results, the vulnerability of the forest ecosystem showed a trend of gradually being aggravated. The FVI value had changed from 0.59 (2000) to 0.67 (2015) (Table 5). Additionally, the *vulnerable*, *highly vulnerable, and extremely vulnerable* levels all showed an increasing trend, with the area proportions increased by 7.44%, 14.20%, and 12.65%, respectively. Inversely, the *slightly vulnerable* and *moderately vulnerable* levels expressed decreasing trends with the area proportions decreased by 6.12% and 28.14%, respectively. However, the spatial distribution of FVI rate of change expressed the heterogeneity of forest vulnerability among the whole area (Figure 8d). Vulnerability changes in Yunnan were relatively weak. However, the forests in southern Tibet showed a trend of increasing vulnerability. The increased and decreased forest lands were alternately distributed in Sichuan Province. Overall, 89% of the forests experienced vulnerability intensification. The spatial change rates of the three vulnerability factors (VNF, VED and VSI) (Figure 8a–c) indicate that the alternating distribution of VSI value has a greater impact on the overall forest vulnerability.

### 3.3. Heterogeneity of Forest Vulnerability under Different Terrains

**Figure 9:**
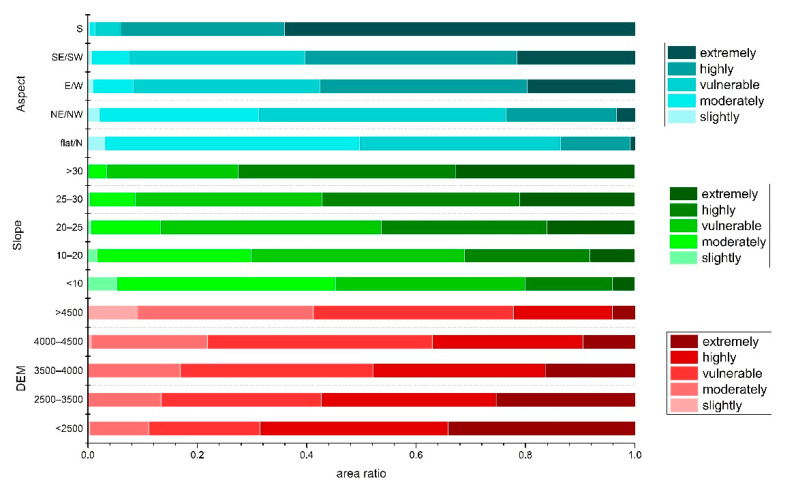
Proportion of different vulnerability levels at different altitudes, slopes, and slopes.

In order to further explore the impact of topography on forest vulnerability, the vulnerability at different altitudes, slopes, and aspects was evaluated. It was observed that most of the forest areas under high and extreme vulnerability came under the elevation ranges of <2500 m and 2500 m–3500 m. A clear tendency can be seen that, as the altitude increased, the ratio of *highly* and *extremely vulnerable* levels gradually decreased, which was opposite to the change of the *slightly* *vulnerable*, *moderately* *vulnerable*, and *vulnerable* levels. High forest vulnerability was observed in steeper areas, while the lower vulnerability was found in relatively flat areas. The proportion of *highly* and *extremely vulnerable* levels increased with improving slope; on the contrary, the ratio of *slightly* and *moderately vulnerable* levels gradually decreased. Besides, the forest vulnerability was more robust in southeast, southwest, and southern aspects. The forest areas under low environmental vulnerability classes were more in the north, northwest, and northeast aspects (Figure 9).

### 3.4. Ecological Protection-Oriented Spatial Pattern of Forest Vulnerability

#### 3.4.1. Strict Protection Region

**Figure 10:**
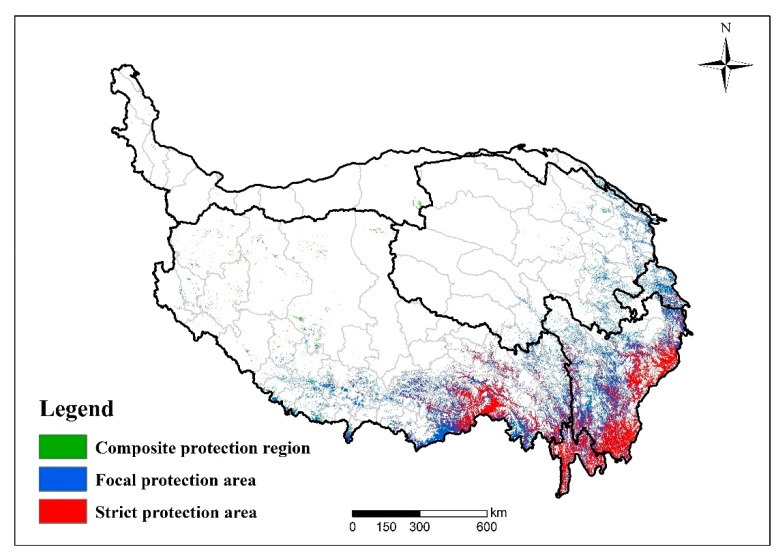
Spatial division of forest ecosystem optimization.

This region was delineated by combining the areas of *highly* and *extremely* vulnerable levels with a positive FVI change rate from 2000 to 2015, and it accounts for approximately 111,078.5 km^2^ (43.5% of the whole forest area). The forest ecosystem in this region was relatively vulnerable, and the degree of vulnerability showed an increasing trend within 15 years. Additionally, the overall ecological potential risk was higher than other areas. This was mainly distributed in the southeastern edge of the QTP (Figure 10).

#### 3.4.2. Focal Protection Region

This region was generated by combining the two kinds of zones that account for 137,453.6 km^2^ of the total area and occupies the highest proportion of forest ecosystems (53.9%). One kind of region was delineated as the levels of *vulnerable*, *highly*, and *extremely* with a negative FVI change rate, indicating that although the forest vulnerability was relatively high, it has shown a mitigatory tendency in recent years. Another kind of area was defined as the vulnerability of *slightly*, *moderately*, and *vulnerable* levels with a positive FVI change rate, indicating that although this area was less vulnerable, it has the potential to intensify its vulnerability (Figure 10).

#### 3.4.3. Composite Protection Region

This region is mainly composed of the *slightly* and *moderately* vulnerable areas, with an FVI change rate of less than zero. This region accounts for approximately 6608.6 km^2^, which is only 2.6% of the whole forest ecosystem. Its distribution is in a few forest plots in western and southern Tibet. These areas are sparsely populated and distributed at high altitudes (Figure 10).

## 4. Discussion

### 4.1. Drivers of Forest Vulnerability Change

Three groups of factors—natural factors, environmental disturbances, and socioeconomic impacts—were synthesized through the distance index model in our research to reflect the comprehensive characteristics of forest vulnerability in the QTP. The background environmental characteristics and socioeconomic impacts of a region had a decisive effect on the vulnerability of the ecosystem [35]. Changes in climate, terrain, and other natural factors have exceeded or changed the threshold of the environmental carrying capacity for human populations and vegetation communities. Additionally, the impact of human activities has gradually increased, which has manifested with the increased potential risk of natural disasters in some areas.

The results of the contribution rate of different factors indicated that the terrain and climate factors are the most important driving factors for VNF (Table 2). During the study period, the proportion of the *highly* and *extremely vulnerable* areas increased, which means the vulnerability has intensified. Taking the small inter-annual changes in topography into account, changes in precipitation and temperature have profoundly affected the development trend of VNF. The climate of the QTP has developed generally in a warm and humid direction [36]. The increase in temperature has caused the melting of glaciers and an increase in runoff [37]. At the same time, the increase in precipitation made the rainfall erosivity more severe [38]. The soil layer was easy to loosen and prone to incur nature disasters such as soil erosion, which puts pressure on the originally complex terrain and ultimately increased the vulnerability. From the results of VED, it can be seen that the vulnerability of the southeastern edge of the QTP was still high. Mountain disasters such as landslides occurred frequently, and climate was still one of the important driving factors. The increases in precipitation and runoff, combined with the steep mountain topography, make the potential energy conditions more forceful, which then gives rise to unstable rocks and soil that slide down, causing collapses and landslides [39,40].

In addition to the background natural factors, the interference of human social and economic activities was also an important driving factor for the escalation of vulnerability. VSI represented the destruction of forests caused by human beings in the process of road and building construction and farmland reclamation, which have all led to a significant increase in forest vulnerability during the study period. Among them, the construction of building lands and the expansion of farmlands were the most important driving factors for the change of VSI (Table 4). In the southeastern part of the QTP, forests were staggered with residential areas and farmland, which deeply affected the integrity of the internal and edge structures of the forest ecosystem and reduced the area of a single forest patch, while increasing the number of patches, leading to serious regional fragmentation [41]. This is also a significant factor affecting the health of forest ecosystems [42]. Simultaneously, artificial surfaces and farmland can reduce the stability of the surrounding ecosystems [43], change geological and hydrological conditions, increase the probability of natural disasters [44,45], which threaten the forest ecosystems that are close to artificial surfaces.

The spatial distribution and change trends of QTP forest vulnerability are the result of multiple driving factors. The FVI integrates a variety of influencing factors, including natural factors, environmental disturbances, and socioeconomic impacts, which can display spatial distributions of forest vulnerability in a comprehensive perspective. Under the integrated drive of multiple factors, the degree of forest vulnerability in different regions and the proportion and change of the five vulnerability levels are different from the results obtained by a single type of driving factor. Therefore, when mitigating vulnerability through forest management, it is necessary to understand the mechanism of each driving factor as well as the mechanism of multiple driving factors. However, it is not enough to make short-term judgments of vulnerability through the calculation of indicators. Long-term classified monitoring and predictions of driving factors are needed to gain a complete understanding of forest vulnerability.

### 4.2. Management Implications for Forest Ecosystem Management in Large Water Tower Regions

The purpose of forest management is to integrate ecology, sociology, management, and other multidisciplinary technologies to maintain forest ecosystems and social stability on a regional scale to obtain sustainable ecosystem products and services [46]. However, as the most important water tower in Asia, the QTP is highly vulnerable to climate change and human activities [47,48], which makes it a challenge to maintain the health and sustainability of forest ecosystems [49]. The vulnerability assessment indicator system of this study fully considered the above two aspects and is an important index to describe the status of forests under climate change and socioeconomic development impacts [50].

Taking the topographic characteristics into account, the vulnerability index interprets the response of the forest ecosystems to the current comprehensive effects of natural contexts, environmental disturbances, and socioeconomic impacts. According to the results of this paper, the ecologically more vulnerable areas should be protected over all others. These vulnerable areas were defined as strict protection regions (Figure 10). During the study period, the main reasons for the increased vulnerability caused by natural contexts were the warm and humid climate conditions and the complicated terrain. Under the combination of climate change and topography, the water retention capacity of forests has also declined [51]. Therefore, the improvement of soil retention capacity is needed in the regions with higher vulnerability [36,52,53]. It is also critical to strengthen the monitoring and prediction of climate changes and consider the impact of climate changes in forest resource assessment, monitoring, investigation, analysis, and long-term planning [54]. In terms of environmental disturbance, the vulnerability tends to decrease, but the vulnerability risk of the southeastern marginal area is still high. Among natural disasters, the landslide factor contributes the most, so the forest vulnerability risk can be reduced by preventing and controlling mountain disasters such as landslides and mudflows [55]. This is also an important part of maintaining the health of forest ecosystems. The disturbance by human activities has greatly increased the vulnerability of forests. Artificial surfaces, including roads, farmlands, and residential areas, have destroyed the integrity of forest ecosystems, especially in areas suitable for human settlements such as the lower altitudes, lower slopes, and southerly regions. In this regard, an ecological security pattern [56] can be constructed through the intervention of forest protection and restoration policies. Improving the protected area systems can be a key approach to limiting human logging and other activities to prevent the ecosystem from losing its resilience [57]. More importantly, the QTP plays an indispensable role in water supply for social economic activities, while the feedback from human activities has increased the QTP’s risk of vulnerability. To adjust the relationship between water yield and forests, it is necessary to balance the development trend of forest habitats from the perspective of vulnerability and the threshold of water ecosystem services that can be provided.

In summary, the forest ecosystems’ vulnerability is an important basis for forest management decision-making [58]. The degree of vulnerability can be used to distinguish the management requirements of forests in different locations [59]. Moreover, the vulnerability can be reduced through targeted forest management programs to adapt to climate change and reduce the interference caused by social and economic development. Additionally, the management of the forest ecosystem on the QTP requires long-term comprehensive monitoring of multiple influencing factors from the perspective of vulnerability to find a balance between forest resources and ecosystem service requirements, which can provide a scientific basis for the sustainable development of mountain forest ecosystems and their service capabilities.

## 5. Conclusions

Forests are crucial ecological components in global water tower regions that provide ecosystem services to human society across local to global scales. QTP is one of the most important water tower regions in Asia, and it is also the largest ecologically vulnerable area in China. The special climate and geological conditions in QTP have bred rich biodiversity and diverse ecosystem services. However, the combined effect of its natural and socioeconomic conditions made the QTP prone to natural disasters, making the forest ecosystem sensitive to environmental changes and likely to lose ecosystem stability.

Using a quantitative forest vulnerability assessment framework based on nature context, environmental disturbances, and socioeconomic impact, this research revealed the spatiotemporal variability of forest vulnerability and its main contributors in the QTP. We found that the measure of vulnerability was unevenly distributed spatially across the study area, and the whole trend has intensified in the past 15 years. The areas that tend to be severe were dominated by steep mountain valleys and rivers that carve down strongly. These areas are prone to natural disasters such as landslides. The factors that contribute the most to the vulnerability of natural contexts, environmental disturbances, and human impacts were slope aspect, landslides, and farmlands, respectively. In addition, the forest vulnerability has obvious distribution patterns with changes in altitude, slope, and aspect. The vulnerability of regions with lower altitudes, steeper slopes, and southerly directions was higher.

According to the FVI index and its change rate, the forest ecosystem of the QTP was divided into three kinds of protection areas: strict protection area, focal protection area, and composite protection area. These vulnerability-based results can be used as an important basis for sustainable forest management in QTP.

## Figures and Tables

**Table 1:** SPCA results of VNF, VED and VSI from 2000 to 2015 (×10^−2^).

PC Layers	Eigenvalue								
	VNF			VED			VSI		
	2000	2010	2015	2000	2010	2015	2000	2010	2015
1	0.58	0.57	0.61	0.27	0.26	0.26	0.11	0.08	0.08
2	0.55	0.52	0.49	0.05	0.06	0.05	0.06	0.05	0.05
3	0.23	0.34	0.27	0.00	0.00	0.00	0.02	0.03	0.03
4	0.17	0.12	0.12						
5	0.11	0.10	0.12						
6	0.11	0.07	0.11						
7	0.07	0.05	0.06						
8	0.03	0.04	0.04						
9	0.03	0.03	0.03						

**Table 2:** The different index contribution of VNF.

Factors	Principal Components									Contribution (%)
	Ⅰ	Ⅱ	Ⅲ	Ⅳ	Ⅴ	Ⅵ	Ⅶ	Ⅷ	Ⅸ	
	Eigenvalue (10^−2^)									
	0.61	0.49	0.27	0.12	0.12	0.11	0.06	0.04	0.03	
	Eigenvectors									
ASPECT	0.6923	0.7187	−0.0633	0.0096	−0.0066	0.0052	−0.0033	0.0022	−0.0035	52.66
FOREST COVERAGE	−0.4490	0.4777	0.5256	0.1596	0.1937	−0.1169	−0.4172	−0.1976	−0.0643	7.15
SOIL OGANIC MATTER	−0.0718	0.0828	0.0806	0.2447	0.1338	−0.7345	0.5773	0.0696	0.1618	1.94
TEMPERATURE	0.2145	−0.1770	0.4096	0.1977	0.0303	−0.0275	−0.1588	0.7939	−0.2424	13.27
WIND SPEED	−0.2585	0.1873	−0.6645	0.3378	0.4556	0.0578	−0.1115	0.2652	−0.2209	−10.13
PERCIPITATION	0.3048	−0.2786	0.2217	0.6000	0.4080	0.3170	0.1207	−0.3623	0.0865	18.33
NPP	−0.2465	0.2554	0.2073	−0.1374	0.0320	0.4829	0.6609	0.0870	−0.3655	7.35
SLOPE	0.1323	−0.1023	0.1057	−0.6168	0.7537	−0.0749	−0.0166	0.0288	0.0751	4.67
DEM	−0.1730	0.1696	0.0256	0.0443	0.0220	0.3217	0.0579	0.3405	0.8458	4.75
Total										100

**Table 3:** The different index contributions of VED.

Factors	Principal Components			Contribution (%)
	Ⅰ	Ⅱ	Ⅲ	
	Eigenvalue			
	0.26	0.05	0.00	
	Eigenvectors			
SOIL EROSION	0.0007	0.0031	0.9999	0.09
FLOOD	0.6620	−0.7495	0.0019	37.63
LANDSLIDE	0.7495	0.6620	−0.0026	62.28
Total				100

**Table 4:** The different index contributions of VSI.

Factors	Principal Components			Contribution (%)
	Ⅰ	Ⅱ	Ⅲ	
	Eigenvalue			
	0.08	0.05	0.03	
	Eigenvectors			
ROAD	0.7136	−0.6559	−0.2463	11.35
FARMLAND	0.5306	0.7355	−0.4212	45.09
BUDING LAND	0.4574	0.1699	0.8729	43.56
Total				100

**Table 5:** Average vulnerability values of the total forest areas in different years.

Vulnerability Index	2000	2010	2015
VNF	0.41	0.45	0.46
VED	0.56	0.52	0.52
VSI	0.89	0.91	0.92
FVI	0.59	0.65	0.67

## Data Availability

Not applicable.
